# Risk factors for recurrent tuberculosis after successful treatment in a high burden setting: a cohort study

**DOI:** 10.1186/s12879-020-05515-4

**Published:** 2020-10-23

**Authors:** Patrick George Tobias Cudahy, Douglas Wilson, Ted Cohen

**Affiliations:** 1 Section of Infectious Disease, Department of Medicine, Yale University School of Medicine, New Haven, CT USA; 2 Department of Internal Medicine, Edendale Hospital, University of KwaZulu-Natal, 5th Floor, Private Bag X 509, Plessislaer, KZN, Pietermaritzburg, 3216 South Africa; 3 Department of Epidemiology (Microbial Diseases), Yale University School of Public Health, New Haven, CT USA

**Keywords:** Mixed-infection, South Africa, Relapse, Reinfection, Fuzzy match

## Abstract

**Background:**

People successfully completing treatment for tuberculosis remain at elevated risk for recurrent disease, either from relapse or reinfection. Identifying risk factors for recurrent tuberculosis may help target post-tuberculosis screening and care.

**Methods:**

We enrolled 500 patients with smear-positive pulmonary tuberculosis in South Africa and collected baseline data on demographics, clinical presentation and sputum mycobacterial cultures for 24-loci mycobacterial interspersed repetitive unit-variable number tandem repeat (MIRU-VNTR) typing. We used routinely-collected administrative data to identify recurrent episodes of tuberculosis occurring over a median of six years after successful treatment completion.

**Results:**

Of 500 patients initially enrolled, 333 (79%) successfully completed treatment for tuberculosis. During the follow-up period 35 patients with successful treatment (11%) experienced a bacteriologically confirmed tuberculosis recurrence. In our Cox proportional hazards model, a 3+ AFB sputum smear grade was significantly associated with recurrent tuberculosis with a hazard ratio of 3.33 (95% CI 1.44–7.7). The presence of polyclonal *M. tuberculosis* infection at baseline had a hazard ratio for recurrence of 1.96 (95% CI 0.86–4.48).

**Conclusion:**

Our results indicate that AFB smear grade is independently associated with tuberculosis recurrence after successful treatment for an initial episode while the association between polyclonal *M. tuberculosis* infection and increased risk of recurrence appears possible.

**Supplementary information:**

**Supplementary information** accompanies this paper at 10.1186/s12879-020-05515-4.

## Introduction

Patients who have recently completed treatment for tuberculosis (TB) are at elevated risk of recurrent TB disease, either as a result of relapse or reinfection [1, 2]. When individuals are diagnosed with recurrent TB, they are less likely to complete treatment and suffer higher mortality than those with first episodes of TB [3]. While there is a growing appreciation of the risks associated with recurrent TB, a clearer picture of which covariates place individuals at greater risk of recurrent disease can inform more targeted post-tuberculosis care and monitoring.

Several covariates have been found to be associated with recurrence of TB after completion of treatment. Drug-resistance [4], smoking [5], HIV infection with low CD4 lymphocyte counts [6], substance use [7], chronic lung disease [8], sputum smear-positive disease [9], and cavitary pulmonary disease [10] have each been found to be associated with increased risk of recurrent tuberculosis. These factors may increase risk of recurrence because they make it more likely for some small numbers of mycobacteria to persist beyond treatment or because they are associated with immune incompetence that places individuals at greater risk of disease following reinfection [11].

Over the last few years, molecular genetic tools have provided a new view on the extent of within-host genotypic heterogeneity of *Mycobacterium tuberculosis* infections. These “mixed-strain” or “heterogeneous” infections arise either through sporadic mutation (clonal heterogeneity) or host acquisition of more than one strain of *M. tuberculosis* (polyclonal heterogeneity), either at the time of infection or through serial re-infection [12]. While detection of mixed-strain infection is limited by the biological samples collected as well as the laboratory processes and sensitivity of sequencing, the frequency at which clonal heterogeneity and polyclonal heterogeneity are reported is nearly 20% in some settings [13]. While several studies have found that within-host heterogeneity, especially polyclonal infections, are associated with poorer treatment response [14], the effects on risk of recurrence are less clear [13].

Studies of recurrent TB are challenging to conduct because the risk of recurrent TB can remain elevated for up to ten years [2]. In this analysis, we combined a research database with electronic notification records to study the long term risk of recurrent tuberculosis among a cohort of ambulatory pulmonary TB patients with a high prevalence of HIV co-infection after successful completion of anti-tubercular therapy.

## Methods

### Setting

The Umgungundlovu health district, in KwaZulu-Natal (KZN), South Africa had a tuberculosis notification rate of 880 cases per 100,000 during the initial study period of 2011. Local HIV prevalence was 16.9% and approximately 70% of people with active tuberculosis were co-infected with HIV [15].

### Participants

All adults with newly diagnosed pulmonary tuberculosis and sputum smear-positive for acid-fast bacilli (AFB) from five primary health care clinics in the Umgungundlovu health district were eligible for the study. Between June 2011 and November 2012 we enrolled 500 participants and collected two spot pre-treatment sputum samples. Immediately after enrollment, participants were initiated on antitubercular therapy according to South African Department of Health guidelines. Further details of study design are available in a previous publication [16].

### Laboratory

Pre-treatment sputum samples were cultured and those positive for *M. tuberculosis* were genotyped with 24-loci mycobacterial interspersed repetitive unit-variable number tandem repeat (MIRU-VNTR) typing at Genoscreen (Institute Pasteur, Lille, France) [17]. Details of the laboratory procedures have been published previously [16]. For samples with multiple repeats at any MIRU-VNTR loci, indicating more than genotype, the ClassTR algorithm [18] was used to distinguish clonal *M. tuberculosis* infection from polyclonal *M. tuberculosis* infection.

### Follow-up

After completion of antitubercular therapy, we tracked outcomes by using routinely collected electronic treatment register (ETR) [19] data. The Umgungundlovu health district ETR data includes 50 clinics serving a population of over 1,000,000 people, and also encompasses the original 5 sites from which participants were recruited [20]. Each facility in the district records tuberculosis case information, including final treatment outcome, in a standardized paper tuberculosis register. The health district collects local facility tuberculosis registers and enters them into a district-wide ETR.

We matched study participants with district data in ETR by exact matching of identifiers (e.g. name, gender, date of birth, treatment start date). As there is no unique patient identifier in South Africa and there are several opportunities during administrative data collection for transcription errors, we conducted fuzzy matching for study participants not located by exact matching. We allowed for substitutions of numerical variables and used Jaro-Winkler [21] distance for matching string variables. A study author (PC) reviewed all potential matches. We recorded the final treatment outcome for each participant. For participants who the ETR listed as lost to follow-up or who transferred their care during treatment, further exact and fuzzy searches were made to attempt to link care episodes and establish a final treatment outcome.

For study participants with documented treatment success (by World Health Organization defined [22] cure or treatment completion) of their initial episode of tuberculosis, a search for secondary episodes of tuberculosis was then made in the electronic register during the period from initial study enrollment though March 30, 2019. For any secondary tuberculosis episodes found in the electronic register, we recorded the time to recurrence from the initial diagnosis as well as the method of tuberculosis diagnosis (empiric, smear microscopy, Gene Xpert MTB/Rif, or culture), and treating clinic.

We constructed Cox proportional hazards models with an outcome of recurrent tuberculosis. Participants were censored on the date the ETR search was made. For a multivariable model, we included covariates for within-host *M. tuberculosis* heterogeneity (simple, clonal, and polyclonal) as this was the focus of the study as well as HIV status, given its strong association with recurrent tuberculosis, and a gaussian frailty random effects term for the health center to adjust for clustering. In addition, covariates with a *p*-value of < 0.20 (age, education, marital status, and AFB sputum smear grade) in univariable models were included.

### Statistical analysis

R statistical software was used to perform statistical analyses [23]. Cox proportional hazard models were constructed using the packages survival [24] version 2.42–6, survminer [25] version 0.4.3, and prodlim [26] version 2018.04.18 to assess whether any factors were associated with recurrent tuberculosis after treatment completion for the initial episode. Proportionality was assessed with correlations between Schoenfeld residuals and log (time) for each covariate and the global model. Multicollinearity was assessed using variance inflation factor.

## Results

**Fig. 1:**
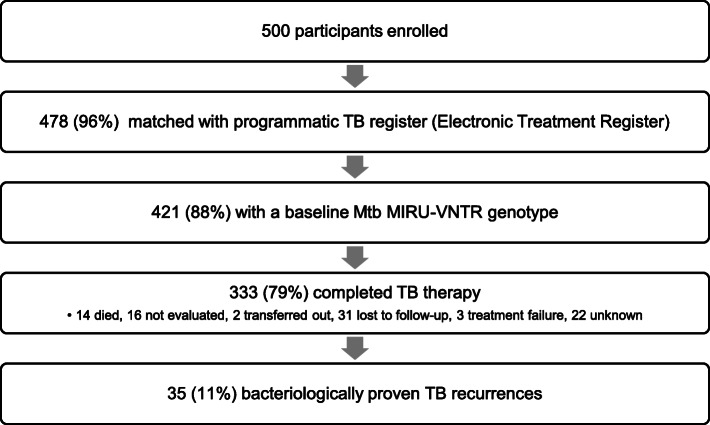
Participant flow diagram

During the study enrollment and follow-up period, 110,859 cases of tuberculosis were recorded in the Umgungundlovu Health District ETR. Of the 500 patients initially enrolled, we matched 478 (96%) within the ETR (Fig. 1). 135 (28%) had a treatment outcome of “not evaluated” due to clinic transfer or loss to follow-up. Further searches allowed discovery of the treating clinic of 89 of these 135 (66%) to determine a final treatment outcome. 421 (88%) of the matched participants had a positive baseline mycobacterial culture with successful MIRU-VNTR genotyping.

333 (79%) of the original study participants with a MIRU-VNTR genotype were recorded as achieving treatment completion or cure for their initial tuberculosis episode (Table 1). Those with a successful treatment outcome were less likely to be living with HIV (65.8 vs 79%) and less likely to have sputum negative for AFB (40.8% vs 56.3%) (Supplemental Table 1). In this cohort, median age was 35 years, and 57% were men. 228 (69%) were HIV positive and 77 (23%) had evidence of heterogeneous *M. tuberculosis* infection by MIRU-VNTR genotyping. When utilizing the ClassTR algorithm to interpret MIRU-VNTR results, 30 (9%) had clonally heterogeneous *M. tuberculosis* disease, and 47 (14%) had polyclonal heterogeneous *M. tuberculosis* disease. 59 (18%) had a history of tuberculosis prior to enrollment. 7 (2%) had multidrug-resistant TB (MDR-TB).

**Table 1:** Baseline characteristics

n	No recurrent tuberculosis	Recurrent tuberculosis
	298	35
Age in years (mean (SD))	35.31 (11.36)	30.03 (8.55)
Male gender (%)	170 (57.0)	21 (60.0)
Married (%)	46 (15.4)	2 (5.7)
Education in years (%)	9.54 (2.63)	9.59 (2.77)
History of imprisonment (%)	18 (6.0)	4 (11.4)
Diabetes (%)	7 (2.3)	2 (5.7)
HIV (%)	206 (69.1)	22 (62.9)
Previous treatment for tuberculosis (%)	54 (18.1)	4 (11.4)
Sputum AFB smear grade		
Scanty (%)	21 (7.0)	2 (5.7)
1+ (%)	69 (23.2)	6 (17.1)
2+ (%)	34 (11.4)	4 (11.4)
3+ (%)	48 (16.1)	13 (37.1)
Clonal mixed TB infection (%)	27 (9.1)	3 (8.6)
Polyclonal mixed TB infection (%)	39 (13.1)	8 (22.9)

**Fig. 2:**
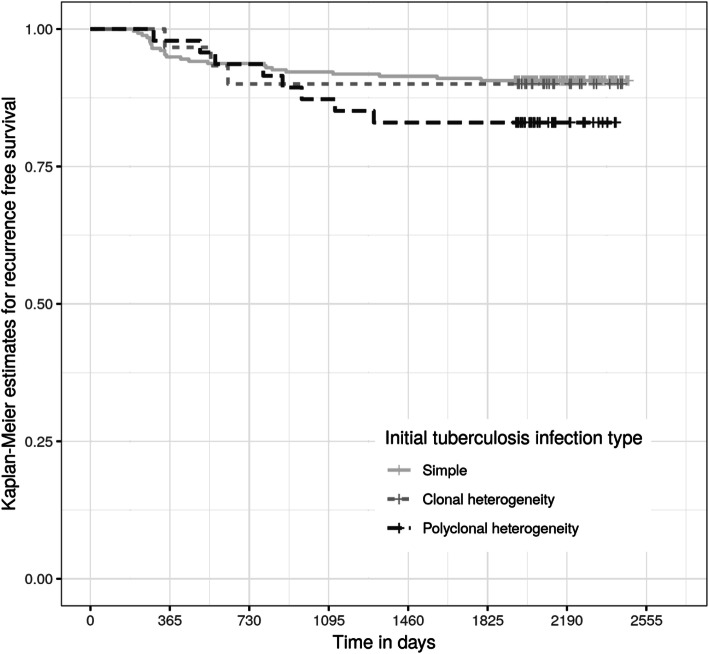
Kaplan Meier estimates for freedom from recurrent tuberculosis after completion or cure

The time from initial tuberculosis diagnosis to searching in the District ETR was a median of 2125 days (interquartile range 2032 to 2271 days). Fourty-five participants (14%) were diagnosed with recurrent tuberculosis, of which 35 participants (11%) had bacteriologically proven tuberculosis, by either sputum smear, Gene Xpert, or culture, after a median of 504 days. Kaplan-Meier curves for bacteriologically proven recurrent tuberculosis after treatment for either simple, clonal or polyclonal *M. tuberculosis* tuberculosis infection were not statistically significantly different by log-rank test (Fig. 2).

**Fig. 3:**
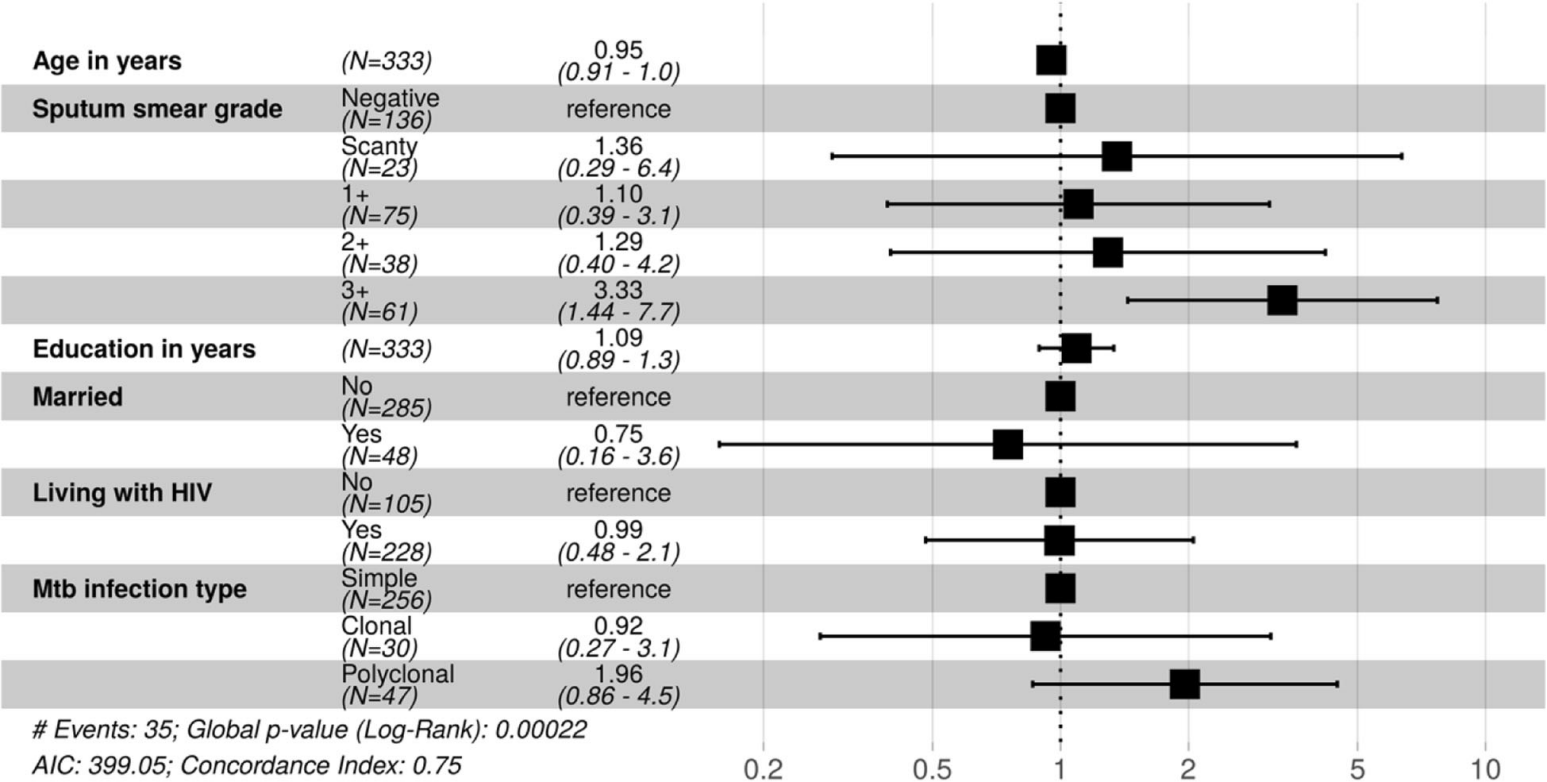
Adjusted hazard ratios for recurrent TB

In univariable analysis with Cox proportional hazards models, younger age was significantly associated with increased risk of bacteriologically proven tuberculosis recurrence with a hazard ratio of 0.94 (95% confidence interval (CI) 0.91–0.98) for every additional year of age (Table 2). In our multivariable model, a 3+ AFB sputum smear grade was significantly associated with recurrent tuberculosis with a hazard ratio of 3.33 (95% CI 1.44–7.7, *p* = 0.005) (Fig. 3). The presence of clonally heterogeneous *M. tuberculosis* infection at baseline had a hazard ratio for recurrence of 0.92 (95% CI 0.27–3.1), while polyclonal heterogeneous *M. tuberculosis* infection at baseline had a hazard ratio for recurrence of 1.96 (95% CI 0.86–4.5). None of those with recurrent tuberculosis had MDR-TB at baseline, which made inclusion of MDR-TB in our models impossible.

**Table 2:** Unadjusted and adjusted hazard ratios for recurrent tuberculosis

n	Unadjusted hazard ratio for recurrent tuberculosis	95% Confidence interval	*p*-value	Adjusted hazard ratio for recurrent tuberculosis	95% Confidence interval	p-value
Age in years	0.95	0.91–0.99	< 0.01	0.91	0.04–1.00	< 0.01
Male gender	1.13	0.57–2.22	0.71			
Married	0.34	0.08–1.45	0.14	0.75	0.16–3.59	0.72
Education in years	1.20	0.99–1.48	0.06	1.09	0.89–1.33	0.40
History of imprisonment	1.92	0.68–5.46	0.22			
Diabetes	2.34	0.56–9.77	0.24			
HIV	0.77	0.39–1.53	0.46	0.99	0.48–2.05	0.99
Previous treatment for tuberculosis	0.59	0.20–1.67	0.32			
Sputum AFB smear grade						
Scanty	1.16	0.26–5.33	0.84	1.36	0.29–6.35	0.70
1+	1.12	0.41–3.10	0.82	1.10	0.39–3.10	0.85
2+	1.47	0.46–4.71	0.51	1.29	0.40–4.20	0.67
3+	3.14	1.38–7.19	< 0.01	3.33	1.44–7.70	< 0.01
Clonal mixed TB infection	1.06	0.32–3.53	0.92	0.92	0.27–3.12	0.90
Polyclonal mixed TB infection	1.82	0.82–3.99	0.14	1.96	0.86–4.48	0.11

## Discussion

After following a cohort of largely HIV-coinfected ambulatory individuals successfully treated for pulmonary tuberculosis disease in a high burden setting for a median of 6 years, we found that 11% (95% CI 7.2 to 13.8%) had bacteriologically proven recurrent tuberculosis and an additional 3% (95% CI 1.2 to 4.8%) had empirically-diagnosed recurrent tuberculosis. This is consistent with other studies performed in high burden settings [2, 27].

Our finding of an association between higher sputum AFB smear grade during the initial tuberculosis episode and recurrent episodes of tuberculosis may be due higher mycobacterial load disease and cavitary disease, leading to a higher risk of relapse after treatment completion. The association between smear grade and poor treatment outcomes, including recurrent disease, has been seen in other studies [28].

In this long-term follow-up, the point estimate for the odds of recurrence for individuals with polyclonal *M. tuberculosis* infection was 1.96, but the 95% confidence interval did not exclude 1. The relatively small number of outcomes recorded limits our ability to make conclusive statements about whether such polyclonal infections make it more likely for patients to experience relapse after successful treatment. An association of recurrent tuberculosis and heterogeneous *M. tuberculosis* infection is plausible as heterogeneous infection leads to the potential for heteroresistance. Additionally, Shin and colleagues recently found an association between heterogeneous *M. tuberculosis* infection and treatment failure in Botswana that was independent of heteroresistance [14]. A high mycobacterial burden within a host or repeated exposures to tuberculosis in a high TB prevalence environment could be other mechanisms for an association of heterogeneous *M. tuberculosis* infection and poor treatment outcomes or recurrence.

HIV co-infection was not associated with the risk of recurrence in univariable or multivariable analysis. This could reflect rising rates of antiretroviral therapy in our setting, and that South African guidelines at the time of our initial study recommended placing patients living with HIV and tuberculosis co-infection on antiretrovirals. Antiretrovirals reduce the risk of developing active tuberculosis, but how that risk compares to the HIV uninfected population is not currently known [6, 29, 30]. Alternatively, participants living with HIV could have suffered higher mortality rates in subsequent years that may have biased our results given we did not have access to vital statistics.

There were limitations in linking our study dataset with ETR data to identify recurrent tuberculosis episodes as there is currently no national health identifier in South Africa. Fuzzy matching of patient identifiers identified many instances of likely matches that were initially missed due to misspellings and transpositions, but we do not know if other matches were missed due to larger discrepancies and whether this introduced bias into our findings. If risk factors of recurrence are also associated with death, lack of access to vital statistics may have biased results by not properly censoring participants who died during the follow-up period.

This limitation leads to our finding of 11% recurrence as a lower bound on the burden of recurrent tuberculosis. Additionally, even though the ETR encompasses a health district that serves over 1,000,000 people, the population of KZN is highly mobile [31] and the ETR database does not capture recurrences outside of the health district. 135 (28%) of the participants in this study transferred clinics or were lost to follow-up during their initial TB treatment and we were able to locate 66% of them at their final treating clinic within the health district. It is conceivable that migration out of the health district may have introduced some bias into our study.

Another limitation is that our sampling was at one timepoint from one anatomical source (sputum). Our group’s work with post-mortem studies in the same population as represented in this study has found significant proportions of multi-organ disseminated tuberculosis with strain heterogeneity that was not captured when sampling sputum alone [32]. Genotyping with MIRU-VNTR also has limited sensitivity for determining *M. tuberculosis* strain diversity and may have underestimated the true proportion of heterogeneous infection [12]. We also were unable to genotype *M. tuberculosis* strains from recurrent episodes to distinguish reinfection from relapse. In other studies with matched fingerprinting of initial and recurrent tuberculosis episodes, reinfection accounted for at least half of recurrent disease, reflecting the hyperendemic communities to which people living with tuberculosis return to after treatment completion. We suspect similarly high rates of reinfection occurred in our population as well, but we do not have genotypes from recurrent tuberculosis episodes to test this hypothesis.

## Conclusions

Our results indicate that AFB smear grade is independently associated with tuberculosis recurrence after successful treatment for an initial episode while the association between polyclonal *M. tuberculosis* infection and increased risk of recurrence appears possible, but further studies are needed to better establish this relationship.

## Supplementary information

**Additional file 1: Supplemental Table 1.** Comparison of participants enrolled but not included in the final analysis vs those included in the final analysis.

## Data Availability

The data sets compiled and analyzed for the current study are available from the corresponding author on reasonable request.

## Abbreviations

AFB: Acid-fast bacilli
CI: Confidence interval
ETR: Electronic treatment register
KZN: KwaZulu-Natal
MDR-TB: Multidrug-resistant tuberculosis
MIRU-VNTR: Mycobacterial interspersed repetitive unit-variable number tandem repeat
TB: Tuberculosis
