# Supplementary material for: Risk factors for recurrent tuberculosis after successful treatment in a high burden setting: a cohort study
Source: BMC Infect Dis. 2020 Oct 23;20:789. doi: 10.1186/s12879-020-05515-4 (PMC7585300; doi:10.1186/s12879-020-05515-4)
Supplement: Supplementary file 1 — Additional file 1: Supplemental Table 1. Comparison of participants enrolled but not included in the final analysis vs those included in the final analysis. [file 12879_2020_5515_MOESM1_ESM.docx]

Supplemental Table 1: Comparison of participants enrolled but not included in the final analysis vs those included in the final analysis

|  |  | Not analyzed | Analyzed | p |
| --- | --- | --- | --- | --- |
| n |  | 167 | 333 |  |
| Age in years (mean (SD)) | | 35.58 (11.74) | 34.75 (11.20) | 0.441 |
| Gender (%) | Women | 79 (47.3) | 142 (42.6) | 0.213 |
|  | Men | 87 (52.1) | 191 (57.4) |  |
|  | NA | 1 (0.6) | 0 (0.0) |  |
| Marital Status (%) | Single | 130 (78.3) | 285 (85.6) | 0.055 |
|  | Married or co-habitating | 36 (21.7) | 48 (14.4) |  |
| Years of education (mean (SD)) | | 9.54 (2.63) | 9.59 (2.77) | 0.858 |
| History of imprisonment (%) | No | 157 (94.6) | 311 (93.4) | 0.749 |
|  | Yes | 9 (5.4) | 22 (6.6) |  |
| Diabetes (%) | No | 162 (97.0) | 324 (97.3) | 0.361 |
|  | Yes | 4 (2.4) | 9 (2.7) |  |
|  | NA | 1 (0.6) | 0 (0.0) |  |
| HIV (%) | No | 29 (17.4) | 108 (32.4) | 0.001 |
|  | Yes | 132 (79.0) | 219 (65.8) |  |
|  | NA | 6 (3.6) | 6 (1.8) |  |
| On HIV anti-retroviral therapy (ART) (%) | No | 139 (83.7) | 297 (89.2) | 0.113 |
|  | Yes | 27 (16.3) | 36 (10.8) |  |
| Previous treatment for tuberculosis (%) | No | 128 (76.6) | 275 (82.6) | 0.127 |
|  | Yes | 38 (22.8) | 58 (17.4) |  |
|  | NA | 1 (0.6) | 0 (0.0) |  |
| Sputum AFB smear grade (%) | Negative | 94 (56.3) | 136 (40.8) | 0.007 |
|  | 1+ | 30 (18.0) | 75 (22.5) |  |
|  | 2+ | 14 (8.4) | 38 (11.4) |  |
|  | 3+ | 19 (11.4) | 61 (18.3) |  |
|  | Scanty | 8 (4.8) | 23 (6.9) |  |
|  | NA | 2 (1.2) | 0 (0.0) |  |
